# Early clinical outcomes after switching to faricimab in previously treated diabetic macular edema

**DOI:** 10.3389/fmed.2026.1920149

**Published:** 2026-09-30

**Authors:** Jing Chen, Bin Chen

**Affiliations:** Department of Ophthalmology, People's Hospital of Leshan, Leshan, Sichuan, China

**Keywords:** anti-VEGF switch therapy, faricimab, macular perfusion, OCT biomarkers, optical coherence tomography angiography, previously treated diabetic macular edema

## Abstract

**Objective:**

To evaluate short-term functional, anatomical, morphologic, and optical coherence tomography angiography (OCTA)-derived outcomes after switching to intravitreal faricimab in previously treated diabetic macular edema (DME) with persistent or recurrent center-involving retinal fluid after conventional anti-vascular endothelial growth factor therapy without angiopoietin-2 inhibition.

**Methods:**

This single-center retrospective study included 50 eyes from 50 patients with previously treated DME and center-involving intraretinal and/or subretinal fluid at the time of switching. All eyes received four consecutive monthly intravitreal faricimab injections. Best-corrected visual acuity (BCVA) and central macular thickness (CMT) were assessed through Month 4. OCT morphologic features were evaluated at baseline and Month 4, and OCTA-derived foveal avascular zone (FAZ) area, superficial capillary plexus vessel density (SCP-VD), and deep capillary plexus vessel density (DCP-VD) were evaluated at baseline, Month 1, and Month 4.

**Results:**

Mean CMT decreased from 526.8 ± 55.8 μm at baseline to 255.6 ± 20.5 μm at Month 4, while mean BCVA improved from 0.80 ± 0.19 to 0.28 ± 0.10 logMAR (both *P* < 0.001), with consistent linear mixed-effects model results. SRF resolved in all 11 eyes with baseline SRF. IRF resolved in 38 of 50 eyes (76.0%) and decreased in the remaining 12 eyes. HRF resolved in 4 of 10 eyes (40.0%) and decreased in the remaining 6 eyes. FAZ area, SCP-VD, and DCP-VD showed no significant longitudinal change. Baseline FAZ area showed weak inverse correlations with CMT reduction and BCVA improvement, but these associations did not remain significant after Bonferroni correction. Baseline SCP-VD and DCP-VD were not significantly associated with treatment response. No serious ocular or systemic adverse events were observed.

**Conclusions:**

Faricimab switching followed by four consecutive monthly injections was associated with substantial short-term anatomical and visual improvement in previously treated DME with persistent or recurrent center-involving retinal fluid. Early clinical improvement occurred without parallel changes in global OCTA-derived perfusion parameters. Larger prospective controlled studies are needed to clarify the treatment-specific contribution and durability of faricimab switching.

## Introduction

Diabetic macular edema (DME) is a major cause of visual impairment in patients with diabetic retinopathy (DR). Its pathogenesis involves chronic hyperglycemia-related inflammation, vascular dysfunction, and breakdown of the blood–retinal barrier (BRB), leading to increased vascular permeability and accumulation of intraretinal and/or subretinal fluid in the macula (1). Intravitreal inhibition of vascular endothelial growth factor (VEGF) is the current first-line treatment for center-involving DME and can substantially reduce macular edema and improve visual acuity in many patients. Nevertheless, a proportion of eyes show persistent or recurrent center-involving retinal fluid after previous anti-VEGF treatment. Such fluid may reflect an inadequate pharmacologic response, but insufficient treatment exposure, prolonged injection intervals, poor systemic control, active proliferative diabetic retinopathy, macular ischemia, and vitreomacular interface abnormalities may produce a similar clinical appearance. Accordingly, eyes with persistent or recurrent retinal fluid after previous anti-VEGF treatment represent a clinically heterogeneous population. In retrospective real-world cohorts, the presence of fluid at a subsequent treatment visit may not always distinguish continuous persistence despite adequately frequent treatment from recurrence after extension of the treatment interval (2, 3).

The mechanisms underlying persistent DME extend beyond VEGF-A-mediated vascular permeability. Angiopoietin-2 (Ang-2), through dysregulation of the Ang/Tie2 pathway, contributes to endothelial destabilization, inflammation, BRB dysfunction, and vascular leakage and may therefore participate in persistent disease activity despite VEGF inhibition (4). Faricimab is a bispecific antibody that simultaneously and independently targets VEGF-A and Ang-2, providing a mechanistically distinct therapeutic approach that may reduce vascular leakage while supporting vascular stability. The phase III YOSEMITE and RHINE trials demonstrated significant visual and anatomical benefits of faricimab in DME, together with the potential for extended treatment intervals (5). However, evidence remains comparatively limited regarding faricimab switching in previously treated eyes with persistent or recurrent DME after conventional anti-VEGF therapy.

Optical coherence tomography angiography (OCTA) provides a noninvasive approach for evaluating macular microvascular characteristics, including the foveal avascular zone (FAZ) and vessel density within the superficial and deep capillary plexuses. Importantly, previous studies have shown heterogeneous OCTA responses after anti-VEGF treatment for DME. In the DOCTA study, improvements in retinal thickness and visual acuity after aflibercept treatment were not accompanied by significant changes in FAZ area, macular perfusion density, or vessel density (6). Similarly, retinal thickness reduction has not consistently correlated with changes in OCTA-derived vessel density, suggesting that resolution of retinal edema and changes in detectable macular perfusion do not necessarily occur in parallel (7). Other prospective data have also shown that treatment-related vessel density changes may vary according to retinal region and that pretreatment OCTA parameters do not consistently predict subsequent visual outcomes (8). Collectively, these findings indicate that structural improvement on optical coherence tomography (OCT) and OCTA-derived microvascular measurements may reflect related but distinct aspects of clinical response.

Whether this relationship is also observed after switching to dual VEGF-A/Ang-2 inhibition in previously treated eyes with persistent or recurrent center-involving DME remains uncertain. Therefore, the present study aimed to characterize the early functional, anatomical, morphologic, and OCTA-derived outcomes after switching to faricimab in eyes with center-involving retinal fluid at the time of switching following previous conventional anti-VEGF therapy without Ang-2 inhibition. Longitudinal changes in best-corrected visual acuity (BCVA) and central macular thickness (CMT) were evaluated together with OCT morphologic responses and selected OCTA-derived microvascular parameters. In addition, exploratory correlation analyses were performed to examine whether baseline FAZ area, superficial capillary plexus vessel density (SCP-VD), and deep capillary plexus vessel density (DCP-VD) were associated with the magnitude of anatomical and visual improvement at Month 4.

## Methods

### Study design and ethical approval

This retrospective, single-center study was conducted at the Department of Ophthalmology, People's Hospital of Leshan. The study adhered to the tenets of the Declaration of Helsinki, as revised in 2024. The study was approved by the Institutional Ethics Committee of the People's Hospital of Leshan [Approval No. LYLL(2026)KY-133], and the requirement for informed consent for the retrospective use of de-identified clinical data was waived by the Ethics Committee. Written informed consent for intravitreal treatment had been obtained from all patients as part of routine clinical care. This study was conducted and reported in accordance with the Strengthening the Reporting of Observational Studies in Epidemiology (STROBE) guidelines (9).

### Participant selection

Medical records of patients with DME who were switched to intravitreal faricimab between January 2024 and December 2025 were retrospectively reviewed. A total of 92 patients (92 eyes) were assessed for eligibility. Of these, 10 eyes were excluded because they did not meet the prespecified anatomical and prior-treatment eligibility criteria or had insufficiently documented previous anti-VEGF treatment, 7 eyes because of incomplete treatment or follow-up data, 12 eyes because of inadequate OCT or OCTA image quality, 5 eyes because of active proliferative diabetic retinopathy requiring additional treatment, and 8 eyes because of significant macular ischemia, vitreomacular interface abnormalities, or other ocular conditions affecting outcome assessment. Finally, 50 eyes from 50 patients were included. Only one eye per patient was included in the analysis. If both eyes met the eligibility criteria, the initial faricimab injections in the two eyes were separated by at least 1 day, and the eye treated earlier was selected as the study eye. All enrolled eyes were switched to intravitreal faricimab therapy and received four consecutive monthly injections in accordance with the regimen recommended in the prescribing information.

### Definition of the study population

Eligible eyes had center-involving DME with intraretinal and/or subretinal fluid involving the foveal center on OCT at the time of switching to faricimab after previous treatment with a standard-dose conventional anti-VEGF regimen consisting of three consecutive monthly injections followed by PRN treatment (3 + PRN). Because retreatment intervals during the subsequent PRN phase were individualized, the retrospective records did not permit a reliable distinction in every eye between fluid that had persisted continuously despite treatment and fluid that had recurred after extension of the treatment interval. Accordingly, the study population is described as previously treated DME with persistent or recurrent center-involving retinal fluid rather than uniformly as refractory DME.

### Inclusion criteria

The inclusion criteria were as follows: (1) age 18 years or older and a diagnosis of type 2 diabetes mellitus; (2) center-involving DME confirmed by clinical examination and OCT; (3) persistent or recurrent center-involving intraretinal fluid and/or subretinal fluid on OCT at the time of switching after prior treatment with a standard-dose conventional anti-VEGF regimen consisting of three consecutive monthly injections followed by PRN treatment (3 + PRN); (4) completion of four consecutive monthly intravitreal faricimab injections; (5) available BCVA data and gradable OCT images at baseline and at Months 1, 2, 3, and 4, together with gradable OCTA images at baseline, Month 1, and Month 4; (6) stable diabetic retinopathy without active retinal neovascularization or a need for additional panretinal photocoagulation or vitreoretinal surgery during follow-up; (7) sufficiently clear ocular media for reliable BCVA, OCT, and OCTA assessment; (8) HbA1c below 7.5%, with blood pressure controlled below 140/90 mmHg in patients with hypertension; and (9) complete clinical, treatment, and imaging records required for the planned analyses.

### Exclusion criteria

The exclusion criteria were as follows: (1) insufficient previous anti-VEGF exposure, lack of documentation confirming the preceding 3 + PRN conventional anti-VEGF treatment course, or incomplete treatment records; (2) active proliferative diabetic retinopathy requiring additional panretinal photocoagulation, vitrectomy, or other treatment; (3) epiretinal membrane, vitreomacular traction, tractional macular edema, or another macular disorder that could affect CMT, retinal fluid, or BCVA; (4) age-related macular degeneration, retinal vein occlusion, retinal detachment, active uveitis, optic nerve disease, uncontrolled glaucoma, or another ocular disease that could affect visual or anatomical outcomes; (5) treatment with a dexamethasone intravitreal implant within 6 months before baseline; (6) treatment with any anti-VEGF agent other than scheduled faricimab, intraocular corticosteroid, macular laser, cataract surgery, or vitreoretinal surgery between baseline and Month 4; (7) corneal opacity, significant lens or posterior capsule opacity, vitreous opacity, or another media opacity affecting examination reliability; (8) OCT or OCTA images with poor signal quality, marked motion or projection artifacts, uncorrectable segmentation errors, or unresolved discrepancies in imaging assessment after independent review by two graders; (9) myocardial infarction, cerebral infarction, or another major cardiovascular or cerebrovascular event within the preceding 3 months, uncontrolled hypertension, or another severe systemic condition that made intravitreal treatment unsuitable; and (10) missing treatment, follow-up, or imaging data required for outcome assessment.

### Outcome measures and imaging assessment

All patients underwent scheduled follow-up examinations at Months 1, 2, 3, and 4 after the initial intravitreal faricimab injection. Best-corrected visual acuity (BCVA), intraocular pressure (IOP), and central macular thickness (CMT) were recorded at baseline and at each follow-up visit using the same examination protocols and instruments throughout the study period. BCVA was measured using a standard decimal visual acuity chart and converted to the logarithm of the minimum angle of resolution (logMAR) scale for statistical analysis. Macular structural imaging was performed using a Cirrus OCT 5,000 system (Carl Zeiss Meditec, Jena, Germany). CMT was automatically measured using the device software at baseline and at Months 1, 2, 3, and 4.

Qualitative optical coherence tomography (OCT) features, including subretinal fluid (SRF), intraretinal fluid (IRF), and hyperreflective foci (HRF), were evaluated at baseline and at Month 4 for the prespecified morphologic outcome analysis. Complete resolution was defined as the absence of the corresponding OCT feature at Month 4 among eyes in which that feature was present at baseline. Reduction was defined as a visible decrease in the corresponding feature at Month 4 compared with baseline without complete disappearance. Optical coherence tomography angiography (OCTA) was performed using a 6 × 6 mm macular scan protocol on the Cirrus OCT 5,000 AngioPlex system (Carl Zeiss Meditec, Jena, Germany). For the planned quantitative OCTA analysis, FAZ area, superficial capillary plexus vessel density (SCP-VD), and deep capillary plexus vessel density (DCP-VD) were assessed at baseline, Month 1, and Month 4. OCTA images were analyzed using the manufacturer's built-in software according to the default segmentation settings. The superficial capillary plexus (SCP) was defined as the layer extending from the internal limiting membrane (ILM) to 10 μm above the inner plexiform layer (IPL), whereas the deep capillary plexus (DCP) was defined as the layer extending from 10 μm above the IPL to 10 μm below the outer plexiform layer (OPL). The foveal region was defined as a circular area with a diameter of 1 mm centered on the fovea. The foveal avascular zone (FAZ) was automatically identified by the OCTA software, and FAZ area, SCP-VD, and DCP-VD were automatically calculated.

All OCT and OCTA images included in the planned analyses were independently reviewed by two authors experienced in retinal imaging. Qualitative OCT features, including SRF, IRF, and HRF, were assessed by both graders. Quantitative OCT and OCTA parameters, including CMT, FAZ area, SCP-VD, and DCP-VD, were obtained from the device software and independently checked for image quality, motion artifacts, and segmentation accuracy. Eyes were included in the final analysis only when the images required for the corresponding prespecified time points were gradable and the imaging assessments were consistent between the two graders or could be resolved by consensus after joint review. Images with poor signal quality, significant motion or projection artifacts, uncorrectable segmentation errors, or unresolved discrepancies after joint review were excluded from analysis.

### Intravitreal faricimab injection procedure

All intravitreal injections were performed by the same experienced retinal specialist under sterile conditions in accordance with the Chinese guidelines for intravitreal injection procedures. Patients received topical levofloxacin eye drops four times daily for 3 days before and after each injection. After standard ocular surface disinfection and sterile draping, topical anesthesia was administered and a lid speculum was inserted. The conjunctival sac was disinfected with 0.5% povidone–iodine for 3 min and then irrigated with sterile saline. Under an operating microscope, the globe was stabilized using forceps, and the injection site was marked 3.5 mm posterior to the limbus in the superior temporal quadrant. A tunneled scleral entry was created by inserting the needle obliquely through the sclera before directing it vertically into the vitreous cavity. Faricimab was administered as a 6-mg intravitreal injection in a volume of 0.05 mL and was injected slowly into the vitreous cavity. Following the injection, light perception was confirmed and antibiotic-steroid ophthalmic ointment was applied to the conjunctival sac. The eye was covered with a sterile dressing for 24 h, after which routine topical medication was resumed.

### Statistical analysis

All statistical analyses were performed using IBM SPSS Statistics for Windows, Version 26.0 (IBM Corp., Armonk, NY, USA). Because only one eye from each patient was included, the study eye was used as the unit of analysis. All 50 study eyes had complete clinical and imaging data required for the planned analyses; therefore, no imputation for missing data was performed.

Continuous variables were assessed for normality using the Shapiro–Wilk test. Normally distributed continuous variables are presented as mean ± standard deviation (SD), with ranges additionally reported where appropriate. Categorical variables are presented as number and percentage [n (%)]. Baseline demographic, systemic, ocular, and previous treatment characteristics were summarized descriptively. Longitudinal changes in central macular thickness (CMT) and best-corrected visual acuity (BCVA) across baseline and Months 1, 2, 3, and 4 were evaluated using one-way repeated-measures analysis of variance (ANOVA). The assumption of sphericity was assessed using Mauchly's test, and the Greenhouse–Geisser correction was applied when the sphericity assumption was violated. Prespecified pairwise comparisons between baseline and each follow-up visit were performed using paired-samples t tests. Because four pairwise comparisons with baseline were performed for each outcome, Bonferroni correction was applied, resulting in an adjusted significance threshold of *P* < 0.0125 for these comparisons. To further account for the correlation among repeated measurements within individual patients, longitudinal changes in CMT and logMAR BCVA were additionally analyzed using linear mixed-effects models. Follow-up time was included as a categorical fixed effect, with baseline specified as the reference time point, and a patient-specific random intercept was included to account for within-patient repeated measurements. Model estimates are reported as β coefficients with standard errors, 95% confidence intervals (CIs), and *P* values.

Longitudinal changes in optical coherence tomography angiography (OCTA)-derived parameters, including foveal avascular zone (FAZ) area, superficial capillary plexus vessel density (SCP-VD), and deep capillary plexus vessel density (DCP-VD), were evaluated at baseline, Month 1, and Month 4 using one-way repeated-measures ANOVA. Sphericity was assessed using Mauchly's test, with Greenhouse–Geisser correction applied when required. Prespecified comparisons of baseline with Month 1 and Month 4 were performed using paired-samples t tests. Because two pairwise comparisons were performed for each OCTA parameter, a Bonferroni-adjusted significance threshold of *P* < 0.025 was applied. Exploratory Pearson correlation analyses were performed to assess the associations of baseline FAZ area, SCP-VD, and DCP-VD with anatomical and visual treatment responses at Month 4. Anatomical response was expressed as *Δ*CMT, calculated as baseline CMT minus Month 4 CMT, such that a larger positive value represented a greater reduction in macular thickness. Visual response was expressed as *Δ*BCVA, calculated as baseline logMAR BCVA minus Month 4 logMAR BCVA, such that a larger positive value represented greater improvement in visual acuity. Pearson correlation coefficients (r) and two-sided *P* values were reported. Because six exploratory correlation tests were performed (three baseline OCTA parameters×two treatment-response outcomes), nominal *P* values were presented, while statistical significance after Bonferroni correction was defined as *P* < 0.0083.

Changes in optical coherence tomography (OCT) morphologic features, including subretinal fluid (SRF), intraretinal fluid (IRF), and hyperreflective foci (HRF), were analyzed descriptively. For each OCT feature, the denominator for the resolution rate was restricted to eyes in which that feature was present at baseline. Outcomes at Month 4 were classified as complete resolution or residual but reduced findings and are presented as *n* (%). No inferential hypothesis testing was performed for these categorical morphologic outcomes. Ocular and systemic adverse events were summarized descriptively as *n* (%). Percentages were calculated using the 50 treated study eyes as the denominator. For each adverse-event category, an eye was counted once regardless of whether repeated episodes of the same event occurred during follow-up. All statistical tests were two-sided. Except where Bonferroni-adjusted thresholds were prespecified for multiple comparisons, a *P* value < 0.05 was considered statistically significant.

## Results

### Baseline characteristics

A total of 50 patients with 50 study eyes were included in the final analysis. All patients had type 2 diabetes mellitus and center-involving DME with intraretinal and/or subretinal fluid at the time of switching after previous conventional anti-VEGF therapy. Before switching to faricimab, the mean number of previous conventional anti-VEGF injections per study eye was 6.0 ± 0.8 (range, 5–7). The mean interval from the last conventional anti-VEGF injection to faricimab initiation was 1.8 ± 0.7 months (range, 1–3 months). Twenty-seven eyes had previously received one conventional anti-VEGF agent, 18 eyes had received two agents, and 5 eyes had received three agents. The last conventional anti-VEGF agent administered before switching to faricimab was ranibizumab in 18 eyes, aflibercept in 10 eyes, and conbercept in 22 eyes. Baseline demographic, systemic, ocular, and prior treatment characteristics are summarized in Table 1.

**Table 1:** Baseline demographic, systemic, ocular, and prior treatment characteristics of the study cohort.

Characteristic	Value
Demographic and systemic characteristics	
Patients (eyes), n	50 (50)
Type 2 diabetes mellitus, *n* (%)	50 (100.0)
Age, years	54.4 ± 8.7 (35–68)
Sex, male/female, n	23/27
Duration of diabetes, years	13.8 ± 3.2 (8–20)
HbA1c, %	6.92 ± 0.43 (6.1–7.4)
Hypertension, *n* (%)	18 (36.0)
Ocular characteristics at baseline	
Duration of DME, months	10.8 ± 2.0 (7–15)
Diabetic retinopathy status, *n* (%)	
Nonproliferative diabetic retinopathy	28 (56.0)
Stable treated proliferative diabetic retinopathy	22 (44.0)
BCVA, logMAR	0.80 ± 0.19
CMT, μm	526.8 ± 55.8 μm
Intraocular pressure, mmHg	15.3 ± 2.1 (11–20)
Center-involving IRF, *n* (%)	50 (100.0)
Center-involving SRF, *n* (%)	11 (22.0)
HRF, *n* (%)	10 (20.0)
Previous conventional anti-VEGF treatment	
Number of previous conventional anti-VEGF injections per study eye	6.0 ± 0.8 (5–7)
Number of previous conventional anti-VEGF agents used, *n* (%)	
One agent	27 (54.0)
Two agents	18 (36.0)
Three agents	5 (10.0)
Last conventional anti-VEGF agent before faricimab, *n* (%)	
Ranibizumab	18 (36.0)
Aflibercept	10 (20.0)
Conbercept	22 (44.0)
Interval from the last conventional anti-VEGF injection to faricimab initiation, months	1.8 ± 0.7 (1–3)

Data are presented as mean ± standard deviation, with ranges provided where applicable, or as *n* (%), unless otherwise indicated. Baseline was defined as the last clinical and imaging assessment performed immediately before the first faricimab injection. All study eyes had previously received conventional anti-VEGF therapy using a 3 + PRN regimen, beginning with three consecutive monthly injections followed by PRN treatment. All patients with hypertension had blood pressure controlled below 140/90 mmHg. BCVA, best-corrected visual acuity; CMT, central macular thickness; DME, diabetic macular edema; HbA1c, glycated hemoglobin; HRF, hyperreflective foci; IRF, intraretinal fluid; logMAR, logarithm of the minimum angle of resolution; PRN, pro re nata; SRF, subretinal fluid; VEGF, vascular endothelial growth factor.

### Longitudinal changes in CMT and BCVA

Longitudinal changes in central macular thickness (CMT) and best-corrected visual acuity (BCVA) are summarized in Table 2. Both anatomical and visual outcomes improved significantly during the 4-month treatment period. Mean CMT decreased from 526.8 ± 55.8 μm at baseline to 355.2 ± 40.7 μm at Month 1, 281.8 ± 30.1 μm at Month 2, 267.8 ± 20.6 μm at Month 3, and 255.6 ± 20.5 μm at Month 4. The overall time effect was significant. Compared with baseline, the mean reductions in CMT were 171.6 μm at Month 1, 245.0 μm at Month 2, 259.0 μm at Month 3, and 271.2 μm at Month 4. All prespecified pairwise comparisons with baseline remained statistically significant after Bonferroni correction (all *P* < 0.001).

**Table 2:** Longitudinal changes in central macular thickness and best-corrected visual acuity during faricimab treatment.

Parameter/time point	Value, mean ± SD	Mean change from baseline	t	*P* value
CMT, μm				
Baseline	526.8 ± 55.8	Reference	—	—
Month 1	355.2 ± 40.7	−171.6	−32.423	<0.001
Month 2	281.8 ± 30.1	−245.0	−38.525	<0.001
Month 3	267.8 ± 20.6	−259.0	−39.136	<0.001
Month 4	255.6 ± 20.5	−271.2	−38.253	<0.001
BCVA, logMAR				
Baseline	0.80 ± 0.19	Reference	—	—
Month 1	0.54 ± 0.14	−0.27	−16.399	<0.001
Month 2	0.45 ± 0.13	−0.35	−17.366	<0.001
Month 3	0.33 ± 0.10	−0.47	−21.329	<0.001
Month 4	0.28 ± 0.10	−0.52	−22.827	<0.001

Data are presented as mean ± standard deviation. Overall longitudinal changes were assessed using one-way repeated-measures analysis of variance. The overall time effect was significant for both CMT F[4,196]=943.360,P<0.001 and BCVA F[4,196]=257.433,P<0.001. Follow-up values were compared with baseline using paired-samples t tests. For the four prespecified pairwise comparisons within each outcome, a Bonferroni-adjusted significance threshold of *P* < 0.0125 was applied. CMT, central macular thickness; BCVA, best-corrected visual acuity; SD, standard deviation.

Mean BCVA improved from 0.80 ± 0.19 logMAR at baseline to 0.54 ± 0.14 at Month 1, 0.45 ± 0.13 at Month 2, 0.33 ± 0.10 at Month 3, and 0.28 ± 0.10 at Month 4. A significant overall time effect was also observed for BCVA. The mean changes from baseline were −0.27, −0.35, −0.47, and −0.52 logMAR at Months 1, 2, 3, and 4, respectively. All pairwise comparisons with baseline remained statistically significant after Bonferroni correction (all *P* < 0.001). To further account for repeated measurements within individual patients, longitudinal changes in CMT and BCVA were additionally evaluated using linear mixed-effects models, as shown in Table 3. With baseline as the reference time point, CMT was significantly reduced at each follow-up visit. At Month 4, the estimated reduction in CMT was 271.2 μm (β = −271.2; 95% CI, −281.3 to −261.1; *P* < 0.001). Similarly, logMAR BCVA was significantly lower at each follow-up visit, with an estimated Month 4 change of −0.52 logMAR (β = −0.52; 95% CI, −0.56 to −0.49; *P* < 0.001). These model-based estimates were consistent with the repeated-measures comparisons presented in Table 2. In a representative eye, serial OCT imaging illustrated progressive resolution of retinal fluid during follow-up, with complete resolution of both SRF and IRF and restoration of the foveal contour by Month 4 (Figure 1).

**Table 3:** Linear mixed-effects model analysis of longitudinal changes in CMT and BCVA.

Outcome	Fixed effect	β coefficient	Standard error	95% CI	*P* value
CMT, μm	Intercept (Baseline)	526.8	5.1	516.9 to 536.7	<0.001
	Time: Month 1	−171.6	5.1	−181.7 to −161.5	<0.001
	Time: Month 2	−245.0	5.1	−255.0 to −234.9	<0.001
	Time: Month 3	−259.0	5.1	−269.0 to −248.9	<0.001
	Time: Month 4	−271.2	5.1	−281.3 to −261.1	<0.001
BCVA, logMAR	Intercept (Baseline)	0.80	0.02	0.76 to 0.84	<0.001
	Time: Month 1	−0.27	0.02	−0.30 to −0.23	<0.001
	Time: Month 2	−0.35	0.02	−0.39 to −0.32	<0.001
	Time: Month 3	−0.47	0.02	−0.50 to −0.43	<0.001
	Time: Month 4	−0.52	0.02	−0.56 to −0.49	<0.001

Linear mixed-effects models included follow-up time as a fixed effect and a patient-specific random intercept to account for repeated measurements. Baseline was used as the reference time point. Negative β coefficients indicate reductions in CMT or logMAR BCVA relative to baseline; for logMAR BCVA, a negative coefficient represents improvement in visual acuity. CI, confidence interval; CMT, central macular thickness; BCVA, best-corrected visual acuity.

**Figure 1:**
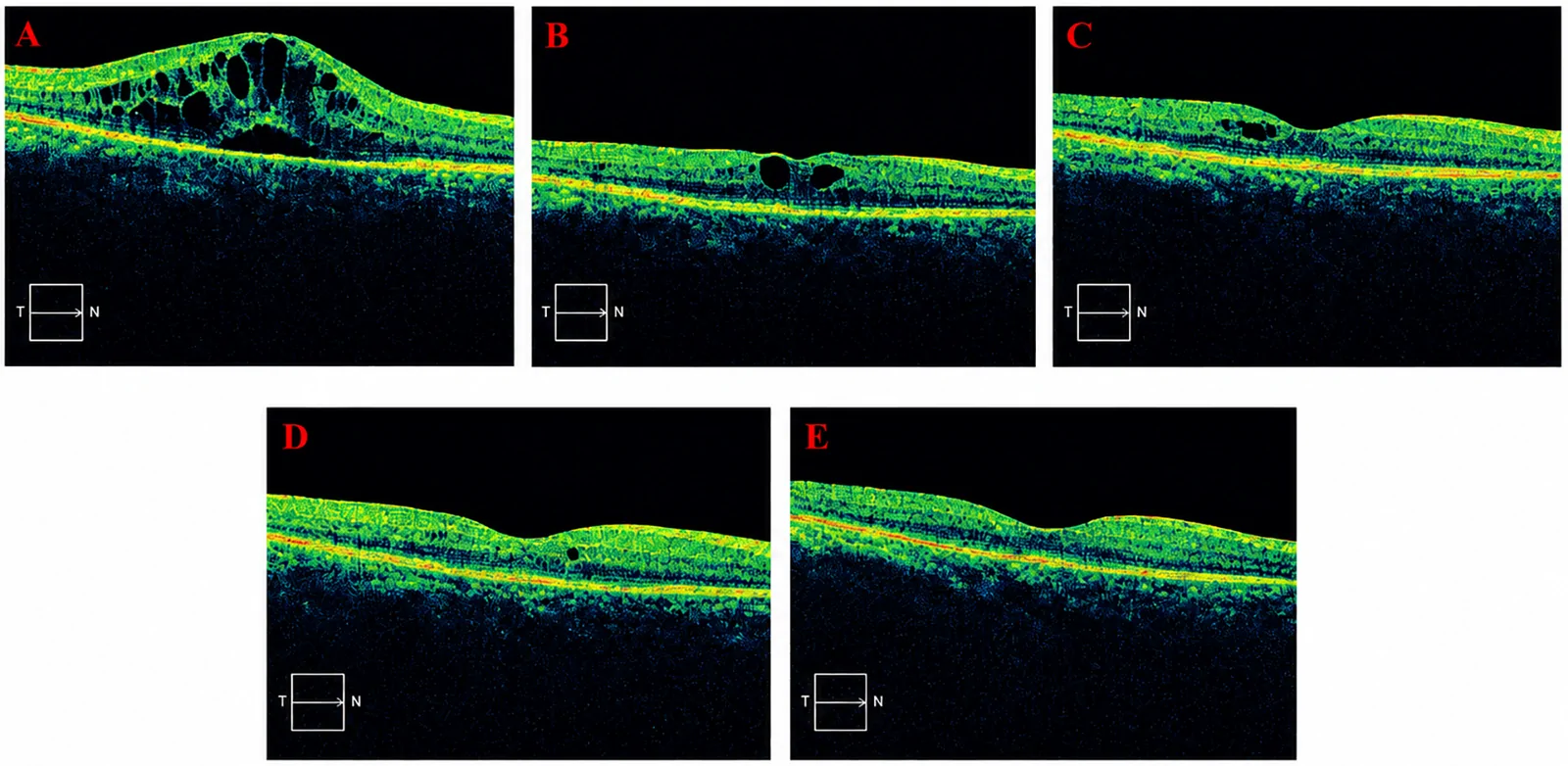
Serial macular optical coherence tomography changes after switching to faricimab in a representative eye with previously treated diabetic macular edema. Representative macular optical coherence tomography (OCT) images of the right eye at baseline and during four months of follow-up after switching to faricimab. Before switching, the eye had received conventional anti-vascular endothelial growth factor (anti-VEGF) therapy using a 3 + PRN regimen. The initial treatment consisted of three consecutive monthly conbercept injections, followed by PRN treatment comprising two ranibizumab injections and one aflibercept injection. The most recent treatment was aflibercept, administered 3 months before baseline. (**A**) At baseline, both intraretinal fluid (IRF) and subretinal fluid (SRF) were present, with marked disruption of the normal foveal contour. (**B**) At Month 1, SRF had resolved and IRF was substantially reduced but remained detectable. (**C**) At Month 2, SRF remained absent, with further reduction of residual IRF. (**D**) At Month 3, SRF remained completely resolved and only minimal residual IRF persisted. (**E**) At Month 4, both SRF and IRF had completely resolved, accompanied by restoration of the foveal contour.

### Changes in OCTA-derived microvascular parameters

Changes in optical coherence tomography angiography (OCTA)-derived microvascular parameters are presented in Table 4. In contrast to the marked improvements in CMT and BCVA, foveal avascular zone (FAZ) area, superficial capillary plexus vessel density (SCP-VD), and deep capillary plexus vessel density (DCP-VD) remained relatively stable during follow-up. Mean FAZ area was 0.337 ± 0.142 mm^2^ at baseline, 0.351 ± 0.151 mm^2^ at Month 1, and 0.345 ± 0.146 mm^2^ at Month 4. No significant overall time effect was observed (F = 0.235, *P* = 0.791). Pairwise comparisons between baseline and Month 1 (*P* = 0.545) and between baseline and Month 4 (*P* = 0.681) were also nonsignificant. Mean SCP-VD was 38.65 ± 4.73% at baseline, 37.98 ± 4.62% at Month 1, and 38.32 ± 4.50% at Month 4. No significant longitudinal change was observed (F = 0.377, *P* = 0.687), and neither the baseline-to-Month 1 comparison (*P* = 0.352) nor the baseline-to-Month 4 comparison (*P* = 0.687) was statistically significant. Mean DCP-VD was 41.39 ± 4.68% at baseline, 40.71 ± 4.79% at Month 1, and 41.04 ± 4.56% at Month 4. The overall time effect was not significant (F = 0.347, *P* = 0.708), and no significant differences were observed between baseline and Month 1 (*P* = 0.371) or between baseline and Month 4 (*P* = 0.689). Thus, none of the three OCTA-derived microvascular parameters showed a statistically significant change during the 4-month observation period.

**Table 4:** Changes in OCTA parameters during faricimab treatment.

Parameter	Baseline	Month 1	Month 4	F	Overall *P* value	P1	P2
FAZ area, mm^2^	0.337 ± 0.142	0.351 ± 0.151	0.345 ± 0.146	0.235	0.791	0.545	0.681
SCP-VD, %	38.65 ± 4.73	37.98 ± 4.62	38.32 ± 4.50	0.377	0.687	0.352	0.687
DCP-VD, %	41.39 ± 4.68	40.71 ± 4.79	41.04 ± 4.56	0.347	0.708	0.371	0.689

Data are presented as mean ± standard deviation. Overall comparisons across baseline, Month 1, and Month 4 were performed using one-way repeated-measures analysis of variance. P1 represents the comparison between baseline and Month 1, and P2 represents the comparison between baseline and Month 4 using paired-samples t tests. For the two prespecified pairwise comparisons within each OCTA parameter, statistical significance was interpreted using a Bonferroni-adjusted threshold of *P* < 0.025. FAZ, foveal avascular zone; SCP-VD, superficial capillary plexus vessel density; DCP-VD, deep capillary plexus vessel density; OCTA, optical coherence tomography angiography.

### Correlation between baseline OCTA parameters and treatment response

The associations between baseline OCTA parameters and anatomical and visual improvement at Month 4 are shown in Table 5. Exploratory Pearson correlation analyses were performed using the reduction in CMT and improvement in logMAR BCVA as measures of treatment response. Baseline FAZ area showed weak inverse correlations with CMT reduction (r = −0.280, nominal *P* = 0.049) and improvement in logMAR BCVA (r = −0.292, nominal *P* = 0.039). These findings indicated that a larger baseline FAZ area tended to be associated with smaller anatomical and visual improvements at Month 4. In contrast, baseline SCP-VD was not significantly associated with either CMT reduction r=0.194,P=0.177 or BCVA improvement r=0.081,P=0.575. Baseline DCP-VD was likewise not significantly correlated with CMT reduction (r = −0.171, *P* = 0.235) or BCVA improvement (r = −0.121, *P* = 0.403). Because six exploratory correlation tests were performed, a Bonferroni-adjusted significance threshold of *P* < 0.0083 was applied. After correction for multiple comparisons, none of the associations shown in Table 5 remained statistically significant.

**Table 5:** Correlation of baseline OCTA parameters with anatomical and visual changes at month 4.

Baseline parameter	*Δ*CMT		*Δ*BCVA	
	*r*	*P* value	*r*	*P* value
FAZ area	−0.280	0.049	−0.292	0.039
SCP-VD	0.194	0.177	0.081	0.575
DCP-VD	−0.171	0.235	−0.121	0.403

Correlations were assessed using Pearson correlation analysis. *Δ*CMT was calculated as baseline CMT minus Month 4 CMT, with a larger positive value indicating a greater reduction in macular thickness. *Δ*BCVA was calculated as baseline logMAR BCVA minus Month 4 logMAR BCVA, with a larger positive value indicating greater visual improvement. *P* values are two-sided. Given the exploratory nature of the analysis, nominal *P* values are presented; after Bonferroni correction for six correlation tests, statistical significance was defined as *P* < 0.0083. FAZ, foveal avascular zone; SCP-VD, superficial capillary plexus vessel density; DCP-VD, deep capillary plexus vessel density; CMT, central macular thickness; BCVA, best-corrected visual acuity; OCTA, optical coherence tomography angiography.

### Changes in OCT morphologic features

OCT morphologic features also improved after four consecutive monthly faricimab injections. Subretinal fluid (SRF) was present in 11 eyes at baseline and completely resolved in all 11 eyes by Month 4, corresponding to a complete resolution rate of 100.0% (11/11). Intraretinal fluid (IRF) was present in all 50 eyes at baseline. Complete IRF resolution was observed in 38 eyes at Month 4, whereas the remaining 12 eyes showed residual but reduced IRF, resulting in a complete resolution rate of 76.0% (38/50). Hyperreflective foci (HRF) were present in 10 eyes at baseline. At Month 4, HRF completely resolved in 4 eyes and decreased in the remaining 6 eyes, corresponding to a complete resolution rate of 40.0% (4/10). These morphologic outcomes are shown in Figure 2.

**Figure 2:**
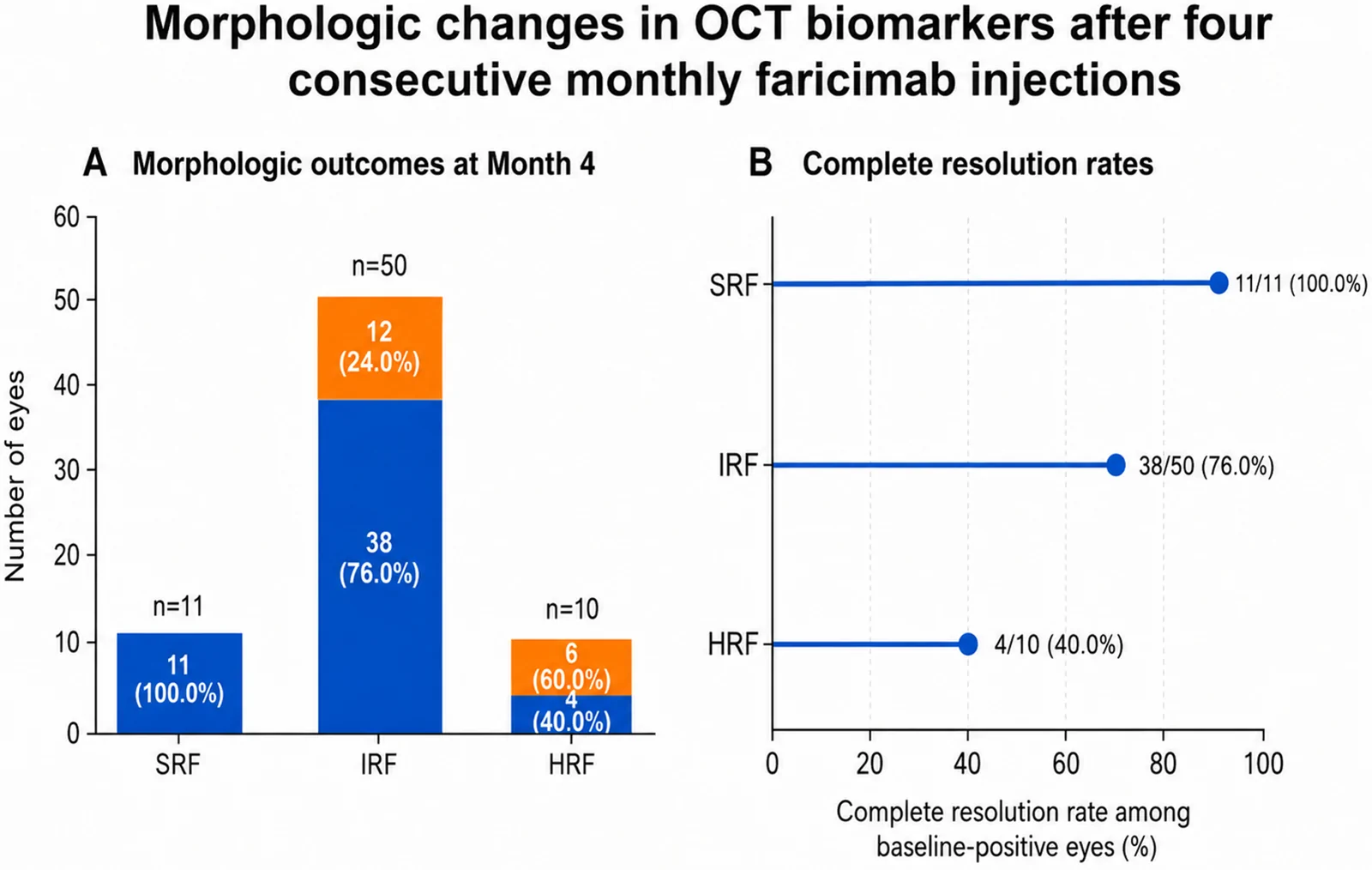
Morphologic changes in OCT biomarkers after four consecutive monthly faricimab injections. Morphologic outcomes were assessed at Month 4 after four consecutive monthly intravitreal faricimab injections in 50 study eyes with previously treated diabetic macular edema and center-involving retinal fluid at the time of switching. In panel **A**, blue/lower segments indicate complete resolution, whereas orange/upper segments indicate residual but reduced findings at Month 4. **(A)** Outcomes of subretinal fluid (SRF), intraretinal fluid (IRF), and hyperreflective foci (HRF) among eyes in which the corresponding feature was present at baseline. SRF was present in 11 eyes at baseline and completely resolved in all 11 eyes (100.0%). IRF was present in all 50 eyes; complete resolution was observed in 38 eyes (76.0%), while 12 eyes (24.0%) showed a reduction with residual IRF. HRF was present in 10 eyes at baseline; complete resolution was observed in 4 eyes (40.0%), while 6 eyes (60.0%) showed a reduction with residual HRF. **(B)** Complete resolution rates at Month 4 among eyes positive for the corresponding OCT feature at baseline: SRF, 11/11 (100.0%); IRF, 38/50 (76.0%); and HRF, 4/10 (40.0%). Resolution rates were calculated using the number of eyes with each respective OCT feature at baseline as the denominator. OCT, optical coherence tomography; SRF, subretinal fluid; IRF, intraretinal fluid; HRF, hyperreflective foci.

### Safety outcomes

The safety analysis included all 50 study eyes, which underwent a total of 200 intravitreal faricimab injections. All recorded ocular adverse events were mild and transient. Transient intraocular pressure elevation occurred in 3 eyes (6.0%), with intraocular pressure increasing to 24–27 mmHg and returning to normal within 1 day without medication. Subconjunctival hemorrhage occurred in 2 eyes (4.0%) and resolved within 1 week. Transient ocular pain or discomfort occurred in 5 eyes (10.0%) and resolved spontaneously, while transient floaters occurred in 2 eyes (4.0%) and resolved within 1 week. No retinal vasculitis, retinal artery or vein occlusion, infectious endophthalmitis, non-infectious intraocular inflammation, myocardial infarction, stroke, or other serious ocular or systemic adverse events were recorded during the 4-month follow-up period. Adverse events are summarized in Table 6.

**Table 6:** Ocular and systemic adverse events during follow-up.

Adverse event	No. of eyes (%)	Management and outcome
Transient intraocular pressure elevation	3 (6.0)	IOP increased to 24–27 mmHg and returned to normal within 1 day without medication
Subconjunctival hemorrhage	2 (4.0)	Injection-related; resolved within 1 week
Transient ocular pain or discomfort	5 (10.0)	Resolved spontaneously
Transient floaters	2 (4.0)	Resolved spontaneously within 1 week

Data are presented as *n* (%). Percentages were calculated based on 50 treated eyes. Each study eye received four consecutive monthly faricimab injections, corresponding to 200 intravitreal injection procedures in total. For each adverse-event category, each eye was counted once, and repeated episodes of the same event in the same eye, if present, were not counted as additional eyes. Transient intraocular pressure elevation was defined as IOP >21 mmHg. IOP, intraocular pressure.

## Discussion

Persistent or recurrent DME after previous conventional anti-VEGF treatment represents a clinically heterogeneous and challenging treatment setting, and switching anti-VEGF therapy is one approach used when center-involving retinal fluid remains present after previous treatment (1–3). Faricimab, through simultaneous inhibition of VEGF-A and Ang-2, provides a biologically plausible switching strategy in this setting (4, 5). In the present study, four consecutive monthly faricimab injections were associated with marked anatomical and functional improvement in previously treated eyes with center-involving retinal fluid at the time of switching. CMT decreased substantially over the 4-month treatment period, accompanied by progressive improvement in BCVA, and these longitudinal changes remained consistent in the linear mixed-effects models. OCT morphologic features also improved, with complete resolution of SRF in all affected eyes, complete resolution of IRF in most eyes, and reduction or disappearance of HRF by Month 4. In contrast, FAZ area, SCP-VD, and DCP-VD remained stable during follow-up despite the concurrent improvements in retinal thickness and visual acuity. Exploratory analyses showed weak inverse associations between baseline FAZ area and both anatomical and visual improvement, but neither association remained significant after correction for multiple comparisons. Baseline SCP-VD and DCP-VD were not significantly associated with treatment response. Taken together, these findings indicate that short-term anatomical and visual improvement during four consecutive monthly faricimab injections may occur without measurable parallel changes in global OCTA-derived macular perfusion parameters.

The anatomical and visual improvements observed in this cohort are broadly consistent with accumulating real-world evidence on switching to faricimab in previously treated DME. Esteban-Floría et al. reported significant improvement in BCVA in previously treated eyes after switching to faricimab, together with a mean reduction in central foveal thickness of 37.8 μm, supporting both functional and anatomical improvement in routine clinical practice (10). Deiters et al. similarly observed a substantial anatomical response in 22 eyes with recalcitrant chronic DME, with central subfield thickness decreasing from 468.5 μm before switching to 339.5 μm after the third faricimab injection. BCVA improved numerically from 0.48 to 0.37 logMAR, although the visual change after the third injection was not statistically significant (11). Of particular relevance to the present four-injection regimen, Tatsumi et al. found that both central retinal thickness and BCVA improved significantly after the third and fourth faricimab injections in eyes with aflibercept-refractory DME, whereas earlier responses were less consistent (12). Not all switch studies, however, have shown significant short-term gains in both anatomical and visual outcomes. Ohara et al. reported a significant extension of the treatment recurrence interval after switching to faricimab, whereas changes in BCVA and CMT at 4 months were not statistically significant (13). Collectively, these studies suggest that anatomical improvement after faricimab switching may be more consistent than the magnitude or timing of short-term visual improvement across previously treated DME cohorts.

The magnitude of BCVA improvement in the present cohort was greater than that reported in several previous switch studies and therefore warrants cautious interpretation. This variability is also illustrated by the systematic review and pooled analysis by Khodor et al. of 29 studies involving 2,128 previously treated eyes with wet age-related macular degeneration, in which central subfield thickness decreased significantly across follow-up intervals, whereas visual acuity did not improve significantly within the first 6 months and showed only modest improvement thereafter (14). Although wet age-related macular degeneration and DME differ substantially in underlying pathology and direct cross-disease comparison is not appropriate, the findings of Khodor et al. provide useful context for the observation that anatomical and short-term functional responses after switching may not occur in parallel. Prior treatment exposure also differed markedly: in the Khodor et al. review, the mean number of previous anti-VEGF injections was 32.7 ± 7.6 among the 17 studies reporting this information, compared with 6.0 ± 0.8 injections in the present cohort. In addition, our cohort had relatively poor baseline visual acuity and substantial baseline retinal thickening, while eyes with significant macular ischemia or other ocular conditions likely to affect visual outcomes were excluded. These cohort characteristics may partly contextualize the comparatively large short-term visual gain observed in the present study. At the same time, the interval between the last conventional anti-VEGF injection and faricimab initiation ranged from 1 to 3 months, after which fixed monthly intravitreal treatment was resumed. Renewed monthly VEGF-A suppression may therefore have contributed to part of the observed anatomical and functional improvement. Direct comparison with cohorts specifically defined as refractory or recalcitrant should also be made cautiously because the present retrospective cohort did not require documentation of continuously persistent fluid during every preceding treatment interval.

The OCT morphologic findings further support a substantial anti-exudative response after switching to faricimab. By Month 4, SRF had completely resolved in all 11 eyes in which it was present at baseline, while IRF completely resolved in 38 of 50 eyes and decreased in the remaining 12 eyes. HRF completely disappeared in 4 of 10 eyes and decreased in the other 6 eyes. These findings indicate that the anatomical response was not limited to a reduction in CMT but was accompanied by improvement in several qualitative OCT features. Although the complete resolution of SRF was clinically notable, the relatively small number of eyes with baseline SRF limits direct comparison of treatment responses between different fluid compartments.

In a recent observational study of aflibercept-resistant DME treated with a four-injection monthly faricimab loading phase, Owlya et al. reported a 22% reduction in total IRF at Month 4, while mean vascular density did not change significantly over the same period (15). This finding is particularly relevant to the present study because it supports the concept that resolution of retinal fluid may occur without a parallel measurable change in OCTA-derived perfusion. HRF should be interpreted more cautiously. HRF have been associated with inflammatory activity and other retinal structural abnormalities in DME, but their biological origin is heterogeneous and may also include retinal exudative material rather than a specific inflammatory process (16, 17). Therefore, the reduction in HRF observed in the present cohort is best regarded as an accompanying morphologic improvement rather than direct evidence of reduced retinal inflammation.

Despite the marked anatomical and functional improvement, FAZ area, SCP-VD, and DCP-VD did not change significantly during follow-up. This apparent dissociation is clinically relevant and should not be interpreted as a contradiction between the OCT and OCTA findings. Similar observations have been reported after anti-VEGF treatment for DME. In the prospective DOCTA study, central retinal thickness and visual acuity improved during 48 weeks of aflibercept treatment, whereas FAZ area, macular perfusion density, and vessel density remained unchanged (6). Santamaría et al. likewise found significant reductions in retinal thickness after anti-VEGF therapy but no consistent association between changes in retinal thickness and vessel density across the superficial or deep capillary plexus (7). Other prospective data have shown regional rather than uniform OCTA changes after treatment and have also questioned the ability of baseline OCTA parameters to predict subsequent visual outcomes (8). In a further sectoral analysis by the same research group, based on a substantially overlapping cohort, only small changes in overall SCP and DCP vessel density were observed after 6 months of anti-VEGF therapy, with a significant change confined to the nasal DCP. Little change was observed in areas characterized by ischemic capillary loss, suggesting that whole-field vessel density measurements may mask localized microvascular responses (18). These findings are particularly relevant to the present study because OCTA-derived vessel density represents detectable flow within a defined retinal slab and can also be influenced by cystic displacement of vessels, segmentation, and other edema-related imaging effects.

Evidence specific to faricimab is also emerging. In patients with refractory DME treated with four monthly faricimab injections, Owlya et al. reported a reduction in intraretinal fluid at Month 4 without a significant change in mean vascular density over the same period (15), closely resembling the pattern observed in the present cohort. Conversely, a small 2025 study using a different OCTA platform and quantitative approach reported improvements in several OCTA-derived parameters, including DCP vessel density and nonperfusion area, during four monthly faricimab injections (19). Taken together, the available evidence suggests that OCTA responses after treatment for DME are heterogeneous and may depend on the vascular region examined, baseline microvascular damage, imaging platform, segmentation approach, and the specific OCTA metric used. From a biological perspective, treatment-related reduction in vascular permeability can lead to rapid fluid resolution and retinal thinning without necessarily restoring capillary networks that have already undergone dropout or structural damage. Therefore, the stable FAZ, SCP-VD, and DCP-VD observed in the present study should not be interpreted, in isolation, as evidence of an inadequate clinical response or as excluding treatment-related vascular effects not captured by global OCTA-derived perfusion metrics. Rather, our findings support the view that resolution of exudative edema and short-term changes in OCTA-derived macular perfusion are related but not necessarily parallel components of treatment response.

The exploratory correlation analysis provided limited evidence that baseline OCTA-derived perfusion parameters were associated with subsequent treatment response. Baseline FAZ area showed weak inverse correlations with both CMT reduction and BCVA improvement at Month 4; however, these associations were based on nominal *P* values and did not remain significant after correction for multiple comparisons. Baseline SCP-VD and DCP-VD were not significantly associated with either anatomical or visual improvement. A larger FAZ may reflect more advanced pre-existing macular capillary nonperfusion and could, in theory, limit the potential for structural or functional recovery, but the present findings are insufficient to establish such a relationship. This cautious interpretation is supported by Kansal et al., who found that pretreatment OCT and OCTA parameters were not associated with final visual acuity after an aflibercept loading phase and did not serve as reliable biomarkers of visual outcome (8). Conversely, a small prospective study of treatment-naïve DME reported a larger baseline DCP, FAZ and lower DCP vessel density in anatomical nonresponders, suggesting that baseline microvascular status may carry prognostic information in some settings (20). However, differences in patient population, treatment exposure, OCTA methodology, and outcome definitions limit direct comparison across studies. Accordingly, the weak FAZ associations observed in the present cohort should be considered hypothesis-generating rather than predictive. Baseline OCTA parameters should not currently be used to select patients for faricimab switching.

Faricimab was generally well tolerated during the short-term follow-up in the present cohort. Across 200 intravitreal injections in 50 eyes, the recorded ocular adverse events were mild and transient, and no cases of retinal vasculitis, retinal vascular occlusion, endophthalmitis, non-infectious intraocular inflammation, or serious systemic adverse events were observed. These findings are consistent with the overall safety profile reported in the YOSEMITE and RHINE trials and with longer-term data from the RHONE-X extension study (5, 21). They are also in line with the real-world series reported by Esteban-Floría et al., in which no adverse events were recorded among 105 faricimab-treated eyes (10). Nevertheless, the present study was not powered to detect rare ocular or systemic complications because of its limited sample size and 4-month follow-up. Accordingly, no new safety signal was observed in this small short-term cohort, but larger studies with longer follow-up remain necessary for a more complete assessment of uncommon adverse events.

Several limitations should be considered when interpreting these findings. First, this was a single-center retrospective study with a relatively small sample size, no concurrent control group, and only 4 months of follow-up. The absence of a comparator is particularly important because the observed improvements cannot be attributed specifically to Ang-2 inhibition. The interval between the last conventional anti-VEGF injection and faricimab initiation ranged from 1 to 3 months, after which all eyes resumed fixed monthly intravitreal treatment. Renewed monthly VEGF-A suppression may therefore have contributed to part of the anatomical and visual improvement observed during follow-up. Accordingly, the effects of dosing frequency, continued VEGF-A suppression, prior treatment exposure, natural variation in disease activity, and regression to the mean cannot be separated from any additional treatment effect specifically attributable to faricimab or Ang-2 inhibition. Second, OCTA measurements were available only at baseline, Month 1, and Month 4 and were obtained using a single device and a 6 × 6 mm macular scan. These measurements may not capture localized or later microvascular changes and may not be directly comparable with values obtained using other scan sizes, devices, segmentation algorithms, or vascular metrics. OCTA assessment in DME is also sensitive to segmentation errors, motion and projection artifacts, signal quality, and distortion of retinal layers by cystic edema, all of which can influence quantitative vessel density and FAZ measurements (22, 23). Although images with poor quality or uncorrectable segmentation errors were excluded and all images were independently reviewed, residual measurement variability cannot be excluded. Third, the correlations between baseline OCTA parameters and Month 4 treatment response were exploratory and involved multiple comparisons. The weak nominal associations observed for FAZ therefore require independent validation and should not be interpreted as predictive effects.

In addition, eyes with significant macular ischemia were excluded, which improved the interpretability of treatment-related changes but limits the generalizability of the findings to patients with more advanced diabetic macular ischemia. Despite these limitations, the study provides a clinically relevant observation: in the short term, BCVA and structural OCT findings may be more responsive indicators of short-term clinical response than global OCTA-derived perfusion measurements in previously treated eyes with DME undergoing faricimab switching. Stable FAZ area or vessel density during early follow-up should therefore not, in isolation, be interpreted as evidence of an inadequate clinical response when visual acuity, retinal thickness, and fluid status show clear improvement. Longer prospective studies with larger samples, appropriate comparator groups, standardized OCTA acquisition, and regional as well as global microvascular analyses are needed to determine whether OCTA can provide additional value for treatment monitoring or patient selection.

## Conclusion

In this single-center retrospective study, switching to faricimab followed by four consecutive monthly injections was associated with substantial short-term anatomical and functional improvement in previously treated eyes with DME and persistent or recurrent center-involving retinal fluid at the time of switching. CMT and BCVA improved progressively, accompanied by marked improvement in OCT fluid and morphologic features. In contrast, FAZ area, SCP-VD, and DCP-VD remained stable during follow-up, and baseline OCTA-derived parameters were not robustly associated with Month 4 anatomical or visual improvement after correction for multiple comparisons. These findings suggest that early resolution of exudative edema and improvement in visual function may occur without measurable parallel changes in global OCTA-derived macular perfusion. Stable OCTA parameters during early follow-up should therefore not, in isolation, be interpreted as evidence of an inadequate clinical response. No new safety signal was identified during the 4-month follow-up. Given the retrospective design, limited sample size, absence of a concurrent comparator, and potential contribution of renewed monthly VEGF-A suppression, these findings should be interpreted cautiously. Larger prospective controlled studies with longer follow-up are needed to clarify the treatment-specific contribution and durability of faricimab switching and to determine whether OCTA provides additional value for treatment monitoring or patient selection.

## Data Availability

The original contributions presented in the study are included in the article/Supplementary Material, further inquiries can be directed to the corresponding author.
